# High Fat Feeding in Mice Is Insufficient to Induce Cardiac Dysfunction and Does Not Exacerbate Heart Failure

**DOI:** 10.1371/journal.pone.0083174

**Published:** 2013-12-18

**Authors:** Robert E. Brainard, Lewis J. Watson, Angelica M. DeMartino, Kenneth R. Brittian, Ryan D. Readnower, Adjoa Agyemang Boakye, Deqing Zhang, Joseph David Hoetker, Aruni Bhatnagar, Shahid Pervez Baba, Steven P. Jones

**Affiliations:** Department of Physiology and Biophysics, Institute of Molecular Cardiology, Department of Medicine, University of Louisville, Louisville, Kentucky, United States of America; National Cheng Kung University, Taiwan

## Abstract

Preclinical studies of animals with risk factors, and how those risk factors contribute to the development of cardiovascular disease and cardiac dysfunction, are clearly needed. One such approach is to feed mice a diet rich in fat (i.e. 60%). Here, we determined whether a high fat diet was sufficient to induce cardiac dysfunction in mice. We subjected mice to two different high fat diets (lard or milk as fat source) and followed them for over six months and found no significant decrement in cardiac function (via echocardiography), despite robust adiposity and impaired glucose disposal. We next determined whether antecedent and concomitant exposure to high fat diet (lard) altered the murine heart’s response to infarct-induced heart failure; high fat feeding during, or before and during, heart failure did not significantly exacerbate cardiac dysfunction. Given the lack of a robust effect on cardiac dysfunction with high fat feeding, we then examined a commonly used mouse model of overt diabetes, hyperglycemia, and obesity (db/db mice). db/db mice (or STZ treated wild-type mice) subjected to pressure overload exhibited no significant exacerbation of cardiac dysfunction; however, ischemia-reperfusion injury significantly depressed cardiac function in db/db mice compared to their non-diabetic littermates. Thus, we were able to document a negative influence of a risk factor in a relevant cardiovascular disease model; however, this did not involve exposure to a high fat diet. High fat diet, obesity, or hyperglycemia does not necessarily induce cardiac dysfunction in mice. Although many investigators use such diabetes/obesity models to understand cardiac defects related to risk factors, this study, along with those from several other groups, serves as a cautionary note regarding the use of murine models of diabetes and obesity in the context of heart failure.

## Introduction

Heart disease is the leading cause of death in the United States. In 2010 [1], the CDC reported that one in three Americans is obese. Based on current trends, over one half of the United States population will be obese by the year 2020. Where once heart disease was mainly associated with the elderly, over-eating and physical inactivity have contributed to earlier onset of the disease. Obesity and diabetes are prominent risk factors for development of heart disease and in order to determine the role these risk factors play in heart disease, various animal models have been employed to recapitulate essential elements of these risk factors. Given the ubiquity of genetically modified mice, many investigators have used diabetic/high-fat fed mouse models to attempt to answer important questions about cardiovascular disease. Some of the most prominent models include leptin receptor deficiency (db/db), streptozotocin treatment, and high fat diet-induced obesity, all of which were used in the present study.

Heart disease is a multifactorial condition with alterations in cellular energy utilization involving multiple pathways including, but not limited to, glycolysis, fatty acid oxidation, gluconeogenesis, glycogen synthesis, and hypertrophy [2,3]. When the heart undergoes a stressful event such as a myocardial infarction, it experiences a shift in energy utilization from fatty acids to one that is more glycolytic. This is evidenced by an increase in glucose transporter 1 (GLUT1) density and a decrease in fatty acid oxidation enzymes [4-6]. In light of this energy shift, it is logical to hypothesize that increased fat intake would have deleterious effects on the heart; however, this has not been the case for many studies. Recent findings report that increased fat intake does not have any negative effect on heart failure progression [7-9]. Moreover, some have shown that increased intake of fat may actually exhibit beneficial effects on the heart during failure [10-12]. Therefore, we sought to determine whether a high fat diet or hyperglycemia was sufficient to produce cardiac dysfunction and/or affect the development of heart failure.

## Methods

High Fat Diet Feeding: Mice were fed either a high fat diet (HFD; 60% cal from fat) or a normal chow diet (ND;10% cal from fat). Both diets were purchased from Research Diets Inc. HFD (Cat #:D12412) consists of 20% protein, 20% carbohydrate, and 60% fat. The ND (D12450B) consists of 20% protein, 70% carbohydrate, and 10% fat. The duration the mice spent on each diet is specified in each methods section. For long-term HFD feeding, 6-week-old male C57BL/6 mice were placed on a HFD (60% cal) derived from either lard (Harlan, #118981; HFDL) or milk (Harlan, #TD09766; HFDM), or low fat diet (LFD) for 30-33 weeks. Cardiac function was assessed after 18 weeks (anesthetized echo) and 28 weeks (conscious echo) of feeding. Mice were allowed to resume normal activity before Dexascan and GTT analyses (see below), which were performed approximately two weeks later.

Glucose tolerance and body composition analysis: In the long-term HFD feeding (mentioned above), adipose tissue content and lean mass was measured using a small animal Lunar PIXIMus X-ray densitometer (i.e. Dexascans). Mice on different diets and treatments were anesthetized with isoflurane and subjected to 5 min Dexascans. To examine the effect of HFD on glucose disposal, we performed glucose tolerance test (GTT). Mice were injected sterile glucose (1mg/g lean mass) and plasma glucose was monitored from the blood samples obtained from the tail vein every 15 min for a period of 180 min using an Aviva Accu-Chek glucometer. 

Myocardial Infarction Studies: Adult (3–4 mo. old) mice were subjected to non-reperfused, *in vivo* coronary ligation to induce heart failure, as described previously [13-17] and in accordance with the University of Louisville Animal Care and Use Committee. Using sterile technique, mice were subjected to a thoracotomy and the left coronary artery visualized and permanently occluded with 7-0 silk suture with the aid of a dissecting microscope. After ligation, the chest and skin were closed using 4-0 silk and polyester sutures respectively. Upon recovery of spontaneous respiration, the intubation tube was removed and mice were allowed to recover in a temperature-controlled area supplemented with 100% oxygen.

Myocardial Ischemia/Reperfusion (MI-R) Studies: Three-month-old male db/db mice and their heterozygous littermates were subjected to *in vivo* coronary artery ischemia–reperfusion as previously described [13,15,18-26]. Briefly, mice were anesthetized with intraperitoneal injections of ketamine hydrochloride (50 mg/kg) and sodium pentobarbital (50 mg/kg). The animals were then attached to a surgical board with their ventral side up. The mice were orally intubated with polyethylene (PE)-60 tubing connected to a mouse ventilator (Harvard Apparatus), and the tidal volume and breathing rate set by standard allometric equations. The mice were supplemented with 100% oxygen via the ventilator side port. Body temperature was maintained between 36.5 and 37.5°C using an electrically controlled heat lamp and rectal probe. A left thoracotomy was performed using a thermal cautery, and the proximal left coronary artery was visualized with the aid of a dissecting microscope and completely occluded for 40 min with 7-0 silk suture mounted on a tapered needle (BV-1, Ethicon). After 40 min, the suture was removed and reperfusion was initiated and visually confirmed. The chest was closed in layers using 4-0 silk suture. The skin was closed using 4-0 nylon suture. Ketoprofen was given as analgesia prior to closing the chest. Upon recovery of spontaneous breathing, mice were removed from the ventilator, extubated, and allowed to recover in a warm, clean cage supplemented with 100% oxygen. 

Transverse Aortic Constriction (TAC) surgery: The TAC surgery was conducted in 3-month-old, male C57BL/6J mice by constriction of the transverse aorta as described [27,28] and in accordance with the University of Louisville Animal Care and Use Committee. Briefly, C57BL/6J mice were anesthetized with ketamine (50 mg/kg, intra-peritoneal) and pentobarbital (50 mg/kg, intra-peritoneal), orally intubated with polyethylene-60 tubing, and ventilated (Harvard Apparatus Rodent Ventilator, model 845) with oxygen supplementation. Mice were maintained under anesthesia with an isoflurane vaporizer (1%) supplemented with 100% oxygen. The aorta was visualized through an intercostal incision and a 7-0 nylon suture was looped around the aorta between the brachiocephalic and left common carotid arteries. The suture was tied around a 27-gauge needle (put adjacent to the aorta) to constrict the aorta to a reproducible diameter. Then the needle was removed, leaving a discrete region of stenosis (TAC mice), and the chest was closed. Mice were extubated upon recovery of spontaneous breathing and were allowed to recover in warm, clean cages supplemented with oxygen. Analgesia (ketoprofen, 5mg/kg, subcutaneous) was given before mice recovered from anesthesia (and by 24 and 48 hours later). Sham age-matched mice were subjected to the same procedure except the suture was only passed underneath the aorta and not tied off. At the end of the study, TAC or Sham operated mice were euthanized and the hearts were rapidly excised and weighed. The hearts were then immediately frozen in liquid nitrogen and stored at -80°C, or perfused and fixed for immunohistochemical analysis. 

Echocardiographic Assessment: Transthoracic echocardiography of the left ventricle was performed as previously described [13-15,17,22], in a blinded fashion, and with adjustments for the Vevo 770 echocardiography system. Body temperature was maintained (36.5-37.5°C) using a rectal thermometer interfaced with a servo-controlled heat lamp. Mice were anesthetized with 2% isoflurane, maintained under anesthesia with 1.5% isoflurane, and examined. The mouse was placed chest up on an examination board interfaced with the Vevo 770. The board was outfitted with EKG electrodes for all limbs. Next, depilatory cream was applied to the mouse’s chest and wiped clean to remove all hair in the area of interest. The 707-B (30MHz) scan head was used to obtain 2D images (100 fps) of the parasternal long axis. M-modes were taken from the same anatomical position. The probe was then rotated to acquire a short axis view of the heart. Beginning at the level of the papillary muscles and moving apically, serial 2D images were taken every millimeter. All measurements were taken by utilizing the Vevo 770’s rail system to maintain probe placement and allow for minute adjustments of position. Left ventricular diameters during diastole (LVIDd) left ventricular diameter during systole (LVIDs) and heart rate (HR) were determined from long axis M-modes. Left ventricular fractional shortening (%FS) was calculated as: [(LVIDd-LVIDs)/LVIDd]*100%. Diastolic and systolic volumes were acquired by applying Simpson’s rule of discs to the serially acquired short axis images. Stroke volume (SV) was calculated as: Diastolic volume - Systolic Volume. Ejection Fraction was calculated as: (SV/Diastolic Volume)*100%. Cardiac output was determined by: SV*HR. Relative wall thickness was calculated as (diastolic posterior wall thickness + diastolic anterior wall thickness)/LVIDd. Prior to acquisition of the Vevo system, the STZ study was conducted on an Acuson Sequoia C512 with a 15L8 scan head. This system was not capable of generating volumetric data, thus, only diameters are provided.

Conscious Echocardiography: Because some investigators have used conscious echocardiography for identifying cardiac dysfunction in high-fat diet mice, a similar approach in an additional cohort of mice was used here. To curb the reflex bradycardia that results from the conscious echo procedure (on the first few exposures of the mice to conscious echocardiography), mice are acclimatized via daily echocardiography. Mice are anesthetized with isoflurane and undergo pectoral depilation. Two days later, the acclimatization period starts, which consists of the investigator grasping the mouse with one hand and placing it into a position that exposes the chest. Pre-warmed echo gel is applied, and the echocardiography probe is positioned. This is done for 2-3 minutes both in the morning and in the afternoon for 5 consecutive days. It is recommended that by Day 3 the investigator try to obtain cine loops of the left ventricle and also obtain M-mode images. This is not only invaluable practice for when the mouse’s heart rates are at a usable level, but it also acclimates the animal to the data acquisition process. By Day 5, heart rate should be >600bpm, and useable echo data can be collected. Long axis views of the left ventricle are obtained along with M-modes. The conscious echocardiography procedure does not allow for the use of the rail system, therefore, Simpson’s rule of discs is not reliable. As a result, the data presented are diastolic and systolic diameters, and, fractional shortening, which was calculated as described above.

Mitochondrial Isolation and Respiration Analysis: To determine the effects of prolonged exposure to HFD on mitochondrial function, mitochondria were isolated from the hearts of mice that were fed either a ND or a HFD for 16 weeks. Mice were anesthetized with 5% isoflurane until unconscious at which time the heart was removed and placed in ice-cold isolation buffer (215 mmol/L mannitol, 75 mmol/L sucrose, 0.1% BSA, 20 mmol/L HEPES, and 1 mmol/L EGTA; pH 7.2). The hearts were homogenized and isolated by differential centrifugation as previously described. Briefly, the homogenate was centrifuged at 1,300 x g for 5 min. Following the first spin the supernatant was placed in a fresh tube and the pellet was resuspended in isolation buffer and centrifuged at 1,300 x *g* for 5 min. The supernatant from the first and second spins were collected in separate tubes and spun at 13,000 x *g* for 10 min. The pellets from both tubes were combined and resuspended in 2 mL of isolation buffer without EGTA. A final centrifugation was performed at 10,000 x g for 10 min. The final mitochondrial pellet was then resuspended in isolation buffer without EGTA to yield approximately 10 mg/mL. The protein concentration was determined using a Bradford protein assay.

Mitochondrial respiration was measured using a Seahorse Biosciences XF24 Flux Analyzer (North Billerica, MA) as previously described [29]. Briefly, 5 μg of mitochondria were suspended in 50 μL KCl-respiration buffer (125 mmol/L potassium chloride, 2 mmol/L magnesium chloride, 2.5 mmol/L potassium phosphate monobasic, and 20 mmol/L HEPES). Next, 50 μL of the resuspended mitochondria (5 μg/50 μL) were added to individual XF microplate wells; each sample was run in triplicate and 4 wells served as temperature controls. Centrifuging the XF plate for 3 minutes at 2000 RPM seeded the mitochondria. Following centrifugation, 450 μL of respiration buffer was added to each well. For the determination of mitochondrial respiration, pyruvate plus malate plus ADP, oligomycin, FCCP, and rotenone plus succinate were injected sequentially through ports A–D, respectively, in the Seahorse Flux Pak cartridges to yield final concentrations of 5 mmol/L (pyruvate), 2.5 mmol/L (malate), 1 mmol/L (ADP), 1 μg/mL (oligomycin), 0.001 mmol/L (FCCP) and 100 nmol/L (rotenone), 10 mmol/L (succinate), respectively.

Streptozotocin Treatment: C57BL/6J (B6) male mice were intraperitoneally injected with either streptozotocin (50 mg STZ/kg body weight) or 0.05 M citrate solution (controls) for five consecutive days. After a total of 16 days from the beginning of injections, blood glucose was assessed. Mice that had blood glucose higher than 300mg/dL were admitted to the study and subjected to echo and TAC.

Mitochondrial Swelling Assay: Mitochondrial sensitivity to calcium induced permeability transition was determined as previously described with modifications [30,31]. Briefly, 200 μL of mitochondria in KCl-respiration buffer (2 mg/mL) + Succinate (10 mmol/L) was loaded onto a 96 well plate and challenged with CaCl_2_ (25 nmol/μg mitochondria). Absorbance was measured every 2 seconds for 600 measurements at 520 nm using a Thermo MultiSkan spectrophotometer.

Statistical Analyses: Results are reported as mean ± S.E. Statistical analysis (GraphPad Prism 5.0) was conducted using student’s *t* test or by one-way ANOVA followed by Newman-Keuls Multiple Comparison Test, when appropriate. Survival analyses were performed by plotting Kaplan-Meier estimators, followed by a log-rank test to determine significance. Differences were considered statistically significant if p ≤ 0.05.

Ethical Statement: All animal procedures were performed in accordance with National Institutes of Health guidelines and approved by the University of Louisville Animal Care and Use Committee.

## Results

### Effects of Extended Duration Feeding and Altered Fat Source for High Fat Diet

**Figure 1:**
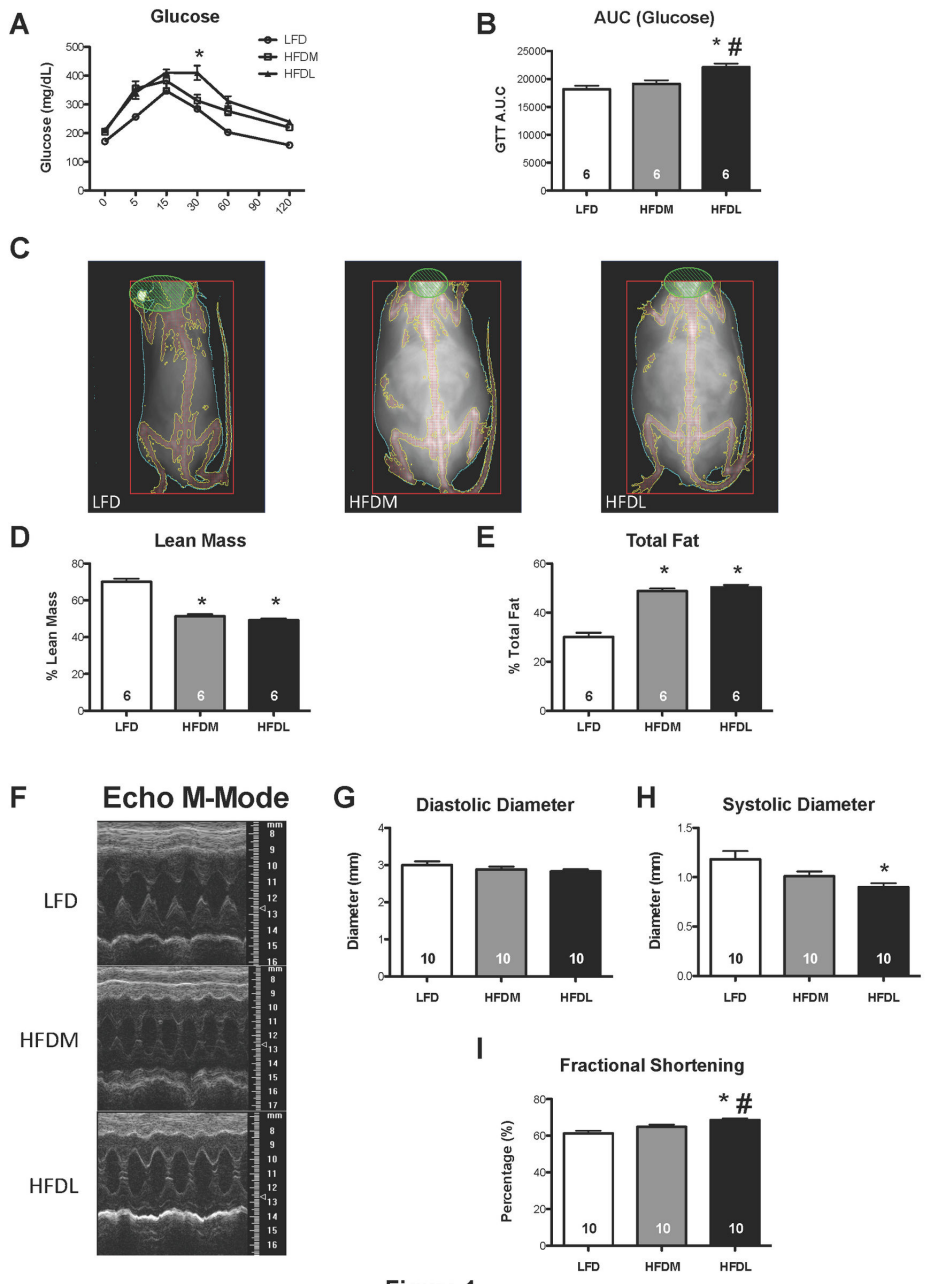
Long-term high fat diet results in altered cardiac function. (**A**) Glucose tolerance test (GTT) (**B**) GTT A.U.C. in LFD, HFDM and HFDL treated mice. (**C**) Dexascan images of LFD, HFDM and HFDL fed mice. (**D**,**E**) Quantitative dexascan analysis of total lean mass and fat mass expressed as percent of total body weight. (**F**) M-mode from conscious echocardiogram utilizing the Vevo 770 imaging system. (**G**-**H**) Diastolic diameter was not significantly altered in either HFD group. Systolic diameter was significantly decreased in HFDL compared to LFD. (**I**) Fractional shortening was significantly elevated in HFDL vs. LFD and HFDL vs. HFDM. Data are presented as mean ± S.E., *p< 0.05 vs. LFD, #p< 0.05 vs. HFDM.

 We investigated whether long-term feeding of high fat diet alone in naïve mice can cause cardiac dysfunction. Recent studies by Russo et al [32] showed that long-term feeding of HFD derived from milk induced cardiac dysfunction(1)(1). Accordingly, we fed 6-week-old male C57/BL6 mice a high fat diet (60% Kcal) of either milk (HFDM) or lard (HFDL), or a low fat diet (LFD). Cardiac function was assessed by anesthetized echocardiography at 18 weeks and by conscious echocardiography at 28 weeks, after starting the diet. No mortalities were seen in any of the groups. After 28 weeks, both the HFDM and HFDL mice weighed significantly more than the LFD group (51.6±0.5 g and 51.9±0.4 g, vs. 34.3±1.0 g, respectively, p0.05 vs. LFD) and Dexascan analysis showed that the percent fat mass was significantly increased and lean mass was significantly reduced in both the HFDM and HFDL compared to LFD fed mice (Figure 1D-E). Glucose tolerance was significantly reduced in the HFDL mice compared with HFDM and LFD fed mice (Figure 1A-B). HFDL mice exhibited higher fractional shortening compared to both LFD and HFDM mice (Figure 1I). The difference in fractional shortening seemed to be largely driven by a reduction in systolic diameter in the HFDL compared to the LFD group (Figure 1G-H). Thus, there was no evidence of cardiac dilation or dysfunction even after over six months of high fat feeding and despite being examined via conscious echocardiography.

### Effects of High Fat Diet on Infarct-Induced Heart Failure

**Figure 2:**
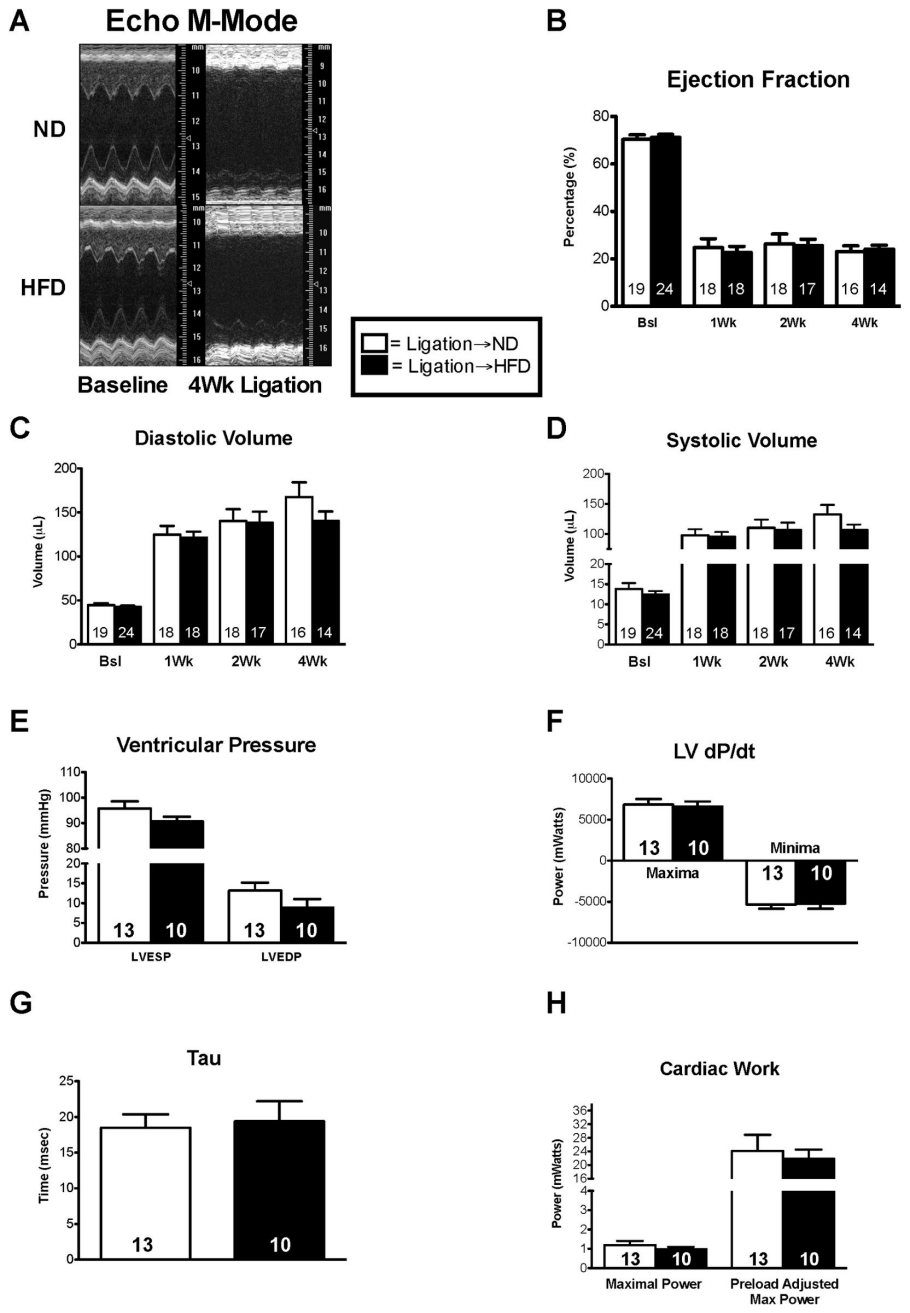
High fat diet post-infarction has no affect on cardiac function. (**A**) Long axis m-mode utilizing the Vevo 770 imaging system. (**B**-**D**) Ejection fraction, diastolic volume, and systolic volume were calculated using Simpson’s method. (**E**) Left Ventricular pressure was assessed via Millar catheter. (**F**) Cardiac contractility as accessed by the minimum and maximum rate of pressure change (dP/dt). (**G**) The rate of relaxation (Tau) and (**H**) the cardiac work remained unchanged between the groups. Results are expressed as means ± S.E., *, p< 0.05.

In order to test the effects of a HFD on the progression of heart failure, 12-week-old C57BL/J6 mice underwent left anterior descending artery ligation and were either placed on a ND (10% fat) or a HFD (60% fat) for a period of 4 weeks. Despite the difference in dietary fat intake and the significant difference in body weight, blood glucose was not significantly altered and there were no significant differences in post-MI mortality, although nominally fewer HFD mice survived (Table 1). Cardiac function was assessed via echocardiography at pre-op, 1, 2, and 4 weeks. Systolic and diastolic volumes were unchanged between the ND and HFD groups (Figure 2C-D). Ejection fraction was also not different between the two groups (Figure 2B). After 4 weeks of coronary ligation, the animals were subjected to pressure volume loop analysis. Both systolic and diastolic pressures were unchanged between the ND and HFD groups (Figure 2E). There were no significant differences in dP/dt or relaxation time (i.e. Tau; Figure 2F-G). Maximal power and preload adjusted maximal power were not different between the groups (Figure 2H).

**Table 1:** Left ventricular diameters, wall thicknesses, and fractional shortening of post-fed HFD group; additionally, body weight, blood glucose, and survival data.

**Time Point**	**Group**	**LVIDd (mm)**	**LVIDs (mm)**	**LVPWd (mm)**	**LVPWs (mm)**	**LVAWd (mm)**	**LVAWs (mm)**	**FS (%)**	**Body Wt (g)**	**Glucose (mg/dL)**	**Survival (Fraction)**
**Pre-Op**	**Lig-ND**	3.6±0.1	2.2±0.1	0.6±0.0	1.0±0.0	0.7±0.0	1.0±0.0	39±1	27.2±1.9	n/a	n/a
	**Lig-HFD**	3.5±0.0	2.2±0.1	0.6±0.0	1.0±0.0	0.7±0.0	1.0±0.0	39±1	26.3±0.5	n/a	n/a
**1 Week**	**Lig-ND**	4.9±0.2	4.3±0.2	0.7±0.0	0.9±0.1	0.7±0.0	0.8±0.0	12±2	n/a	n/a	n/a
	**Lig-HFD**	4.9±0.1	4.4±0.2	0.7±0.0	0.9±0.0	0.7±0.0	0.8±0.0	10±1	n/a	n/a	n/a
**2 Week**	**Lig-ND**	5.1±0.2	4.5±0.2	0.8±0.0	0.9±0.1	0.8±0.0	0.8±0.0	12±2	n/a	n/a	n/a
	**Lig-HFD**	5.1±0.2	4.6±0.2	0.7±0.1	0.9±0.0	0.7±0.0	0.8±0.0	11±1	n/a	n/a	n/a
**4 Week**	**Lig-ND**	5.6±0.2	5.0±0.2	0.7±0.0	0.8±0.1	0.7±0.0	0.8±0.0	11±1	25.4±0.8	152±5	16/19
	**Lig-HFD**	5.3±0.2	4.7±0.2	0.7±0.0	0.9±0.1	0.7±0.0	0.8±0.0	12±1	30.1±1.0*	148±6	14/24

* p<0.05 vs. Lig-ND

### Exposure to High Fat Diet Prior to Infarction does not alter Progression of Heart Failure

**Figure 3:**
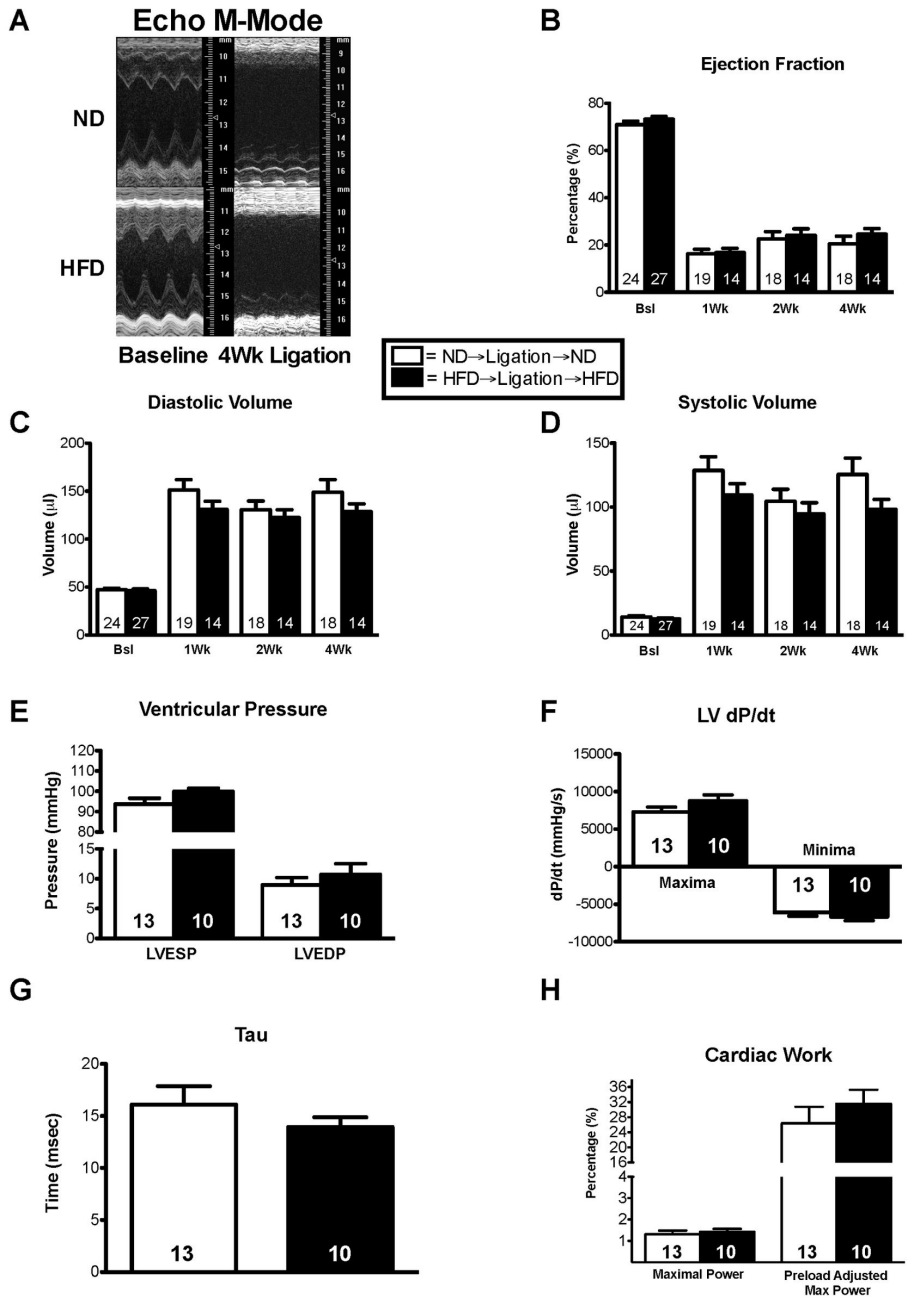
A high fat diet preceding and continued after infarction has no affect on cardiac function. (**A**) Long axis m-mode utilizing the Vevo 770 imaging system. (**B**-**D**) Ejection fraction, diastolic volume, and systolic volume were calculated using Simpson’s method. (**E**) Left Ventricular pressure was assessed via Millar catheter. (**F**) Cardiac contractility as accessed by the minimum and maximum rate of pressure change (dP/dt). (**G**) The rate of relaxation (Tau) and (**H**) the cardiac work remained unchanged between the groups. Results are expressed as means ± S.E., *, p< 0.05.

After finding no functional differences between infarcted mice that were fed a HFD and those that were fed a ND, we tested the hypothesis that mice fed a HFD before infarction would have worse cardiac function than ND fed mice. Therefore, we exposed mice to either a HFD or ND for 6 weeks, after which we subjected all the mice to coronary ligation. The mice were maintained on their respective diets following ligation. Cardiac function was assessed via echocardiography at pre-op, 1, 2, and 4 weeks post-op. There was no significant difference in mortality between the groups during the 4-week study, and despite elevated body weight, blood glucose levels were not different (Table 2). Pre-fed HFD mice had similar systolic and diastolic volumes throughout the 4 weeks of ligation compared to controls (Figure 3C-D). As expected, both groups exhibited dramatic changes in ejection fraction after ligation, however there was no difference between the groups (Figure 3B). After 4 weeks of ligation, the mice were subjected to PV loop analysis. Left ventricular end systolic and diastolic pressures did not change between the HFD and ND groups (Figure 3E). LV dP/dt, Tau, maximal power, and preload adjusted maximal power were not significantly different (Figure 3F-H).

**Table 2:** Left ventricular diameters, wall thicknesses, and fractional shortening of the pre-fed HFD group; additionally, body weight, blood glucose, and survival data.

**Time Point**	**Group**	**LVIDd (mm)**	**LVIDs (mm)**	**LVPWd (mm)**	**LVPWs (mm)**	**LVAWd (mm)**	**LVAWs (mm)**	**FS (%)**	**Body Wt (g)**	**Glucose (mg/dL)**	**Survival (Fraction)**
**Pre-Op**	**ND-Lig-ND**	3.7±0.1	2.3±0.1	0.6±0.0	1.0±0.0	0.7±0.0	0.9±0.0	39±1	27.8±0.7	n/a	n/a
	**HFD-Lig-HFD**	3.7±0.1	2.2±0.0	0.6±0.0	1.1±0.0	0.7±0.0	0.9±0.0	40±1	31.1±0.8*	n/a	n/a
**1 Week**	**ND-Lig-ND**	5.2±0.2	4.6±0.2	0.6±0.0	0.9±0.1	0.7±0.0	0.8±0.0	12±1	n/a	n/a	n/a
	**HFD-Lig-HFD**	4.75±0.1	4.1±0.1	0.7±0.0	0.9±0.0	0.7±0.0	0.8±0.0	13±1	n/a	n/a	n/a
**2 Week**	**ND-Lig-ND**	5.0±0.2	4.4±0.2	0.6±0.0	0.8±0.1	0.7±0.0	0.8±0.0	12±2	n/a	n/a	n/a
	**HFD-Lig-HFD**	5.1±0.1	4.5±0.1	0.6±0.1	0.9±0.1	0.8±0.0	0.9±0.1	12±1	n/a	n/a	n/a
**4 Week**	**ND-Lig-ND**	5.4±0.2	4.8±0.2	0.6±0.0	0.8±0.1	0.8±0.0	0.9±0.1	16±4	27.7±0.5	178±6	18/24
	**HFD-Lig-HFD**	4.9±0.2	4.4±0.2	0.7±0.0	0.8±0.0	0.8±0.0	1.0±0.1	12±1	30.3±1.1*	165±7	14/27

* p<0.05 vs. Lig-ND

### Effects of High Fat Diet on Mitochondrial Respiration and Swelling

**Figure 4:**
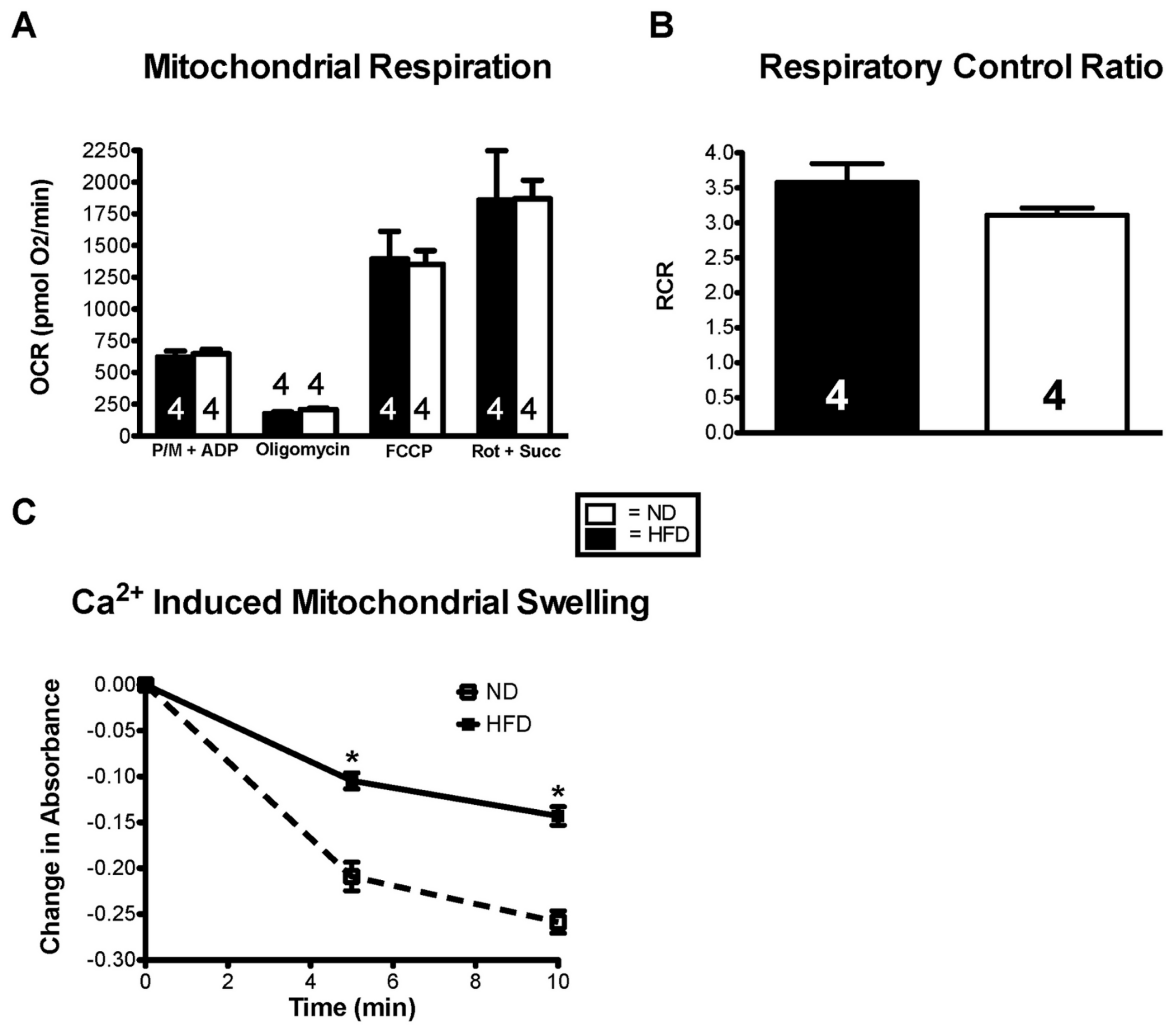
High fat diet does not affect mitochondrial bioenergetics but desensitizes mitochondria to Ca^2+^ induced permeability transition. Mitochondrial bioenergetics were assessed in HFD and ND mice, (**A**) no significant difference in the individual mitochondrial respiration states or (**B**) respiratory control ratios were observed. Mitochondrial swelling following administration of a single bolus of Ca^2+^ was assayed and revealed (**C**) that HFD resulted in a significant increase in mitochondrial Ca^2+^ buffering capacity when compared to ND. Results are expressed as means ± S.E., *, p< 0.05.

To determine the effects of prolonged exposure to a diet high in fat on mitochondrial function, mitochondria were isolated from the hearts of ND and HFD mice. Complex I (state III; pyruvate/malate-driven), complex I maximal (state V-I) and complex II maximal (state V-II; succinate-driven) respiration did not differ between the HFD and ND groups. Likewise, the respiratory control ratio (RCR; a metric of the coupling of electron transport with ATP production) did not differ between groups, indicating a similar level of mitochondrial coupling (Figure 4A-B).

 We also assessed the sensitivity of mitochondria to calcium-induced permeability transition (mPT) by measuring changes in absorbance at 520 nm. Mitochondrial swelling as a result of mPT is detected as a decrease in absorbance. Mitochondria from mice fed a HFD significantly resisted calcium-induced mPT compared to ND mitochondria (Figure 4C). Because we observed no differences in a metric as sensitive as mitochondrial function, we predict that a HFD for 16 wks (or less) would have no impact on heart failure in mice.

### Hyperglycemia Exacerbates I/R-, but not Pressure Overload-, Induced Dysfunction

**Figure 5:**
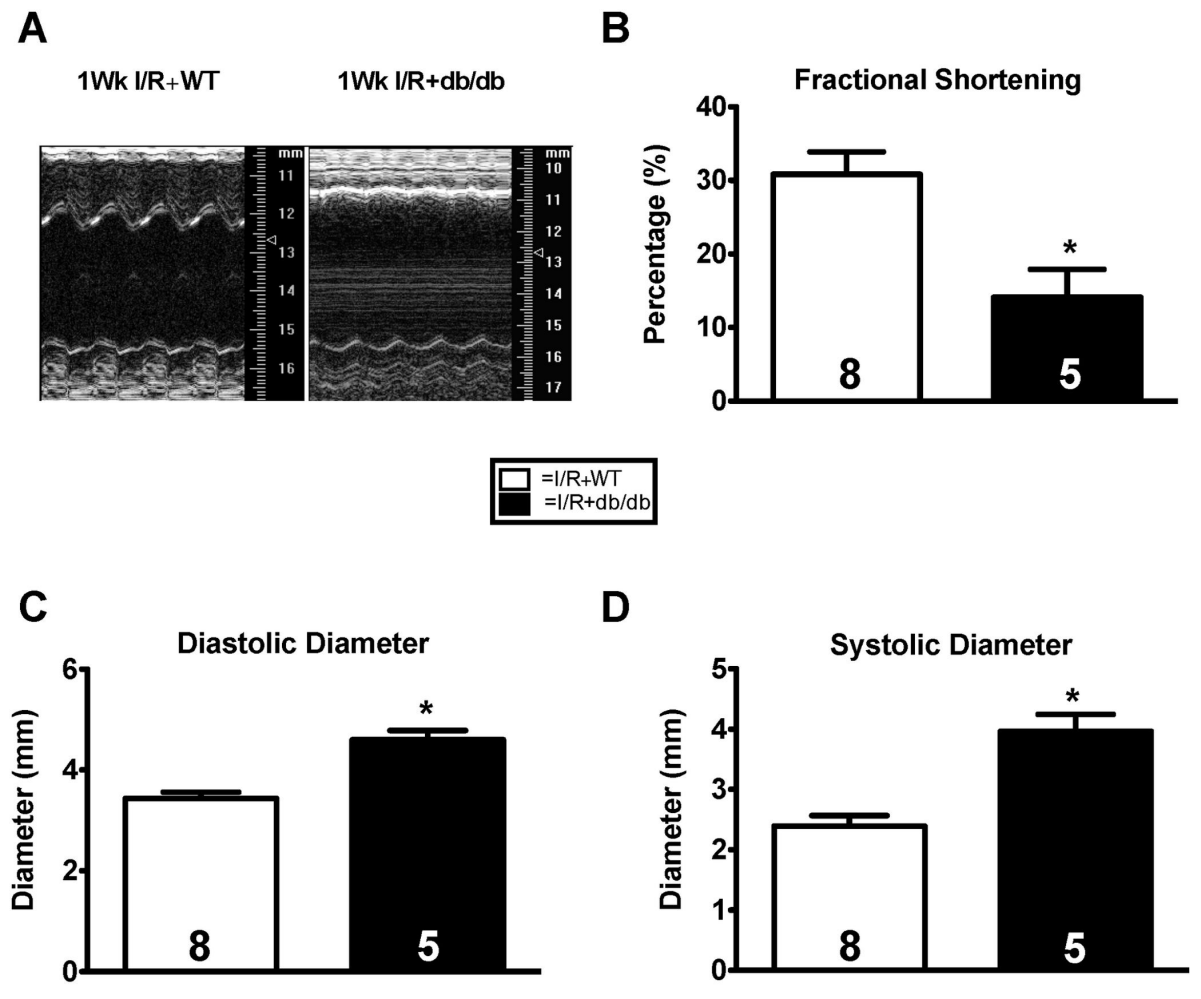
Ischemia reperfusion injury is exacerbated by hyperglycemia and obesity. db/db or heterozygous nondiabetic littermates were subjected to ischemia/reperfusion injury. (**A**) Long axis m-mode utilizing the Vevo 770 imaging system. (**B**-**D**) Fractional shortening was significantly decreased while diastolic diameter and systolic diameter were significantly increased in the IR+db/db group compared to the IR+nondiabetic group. Results are expressed as means ± S.E., *, p< 0.05.

**Figure 6:**
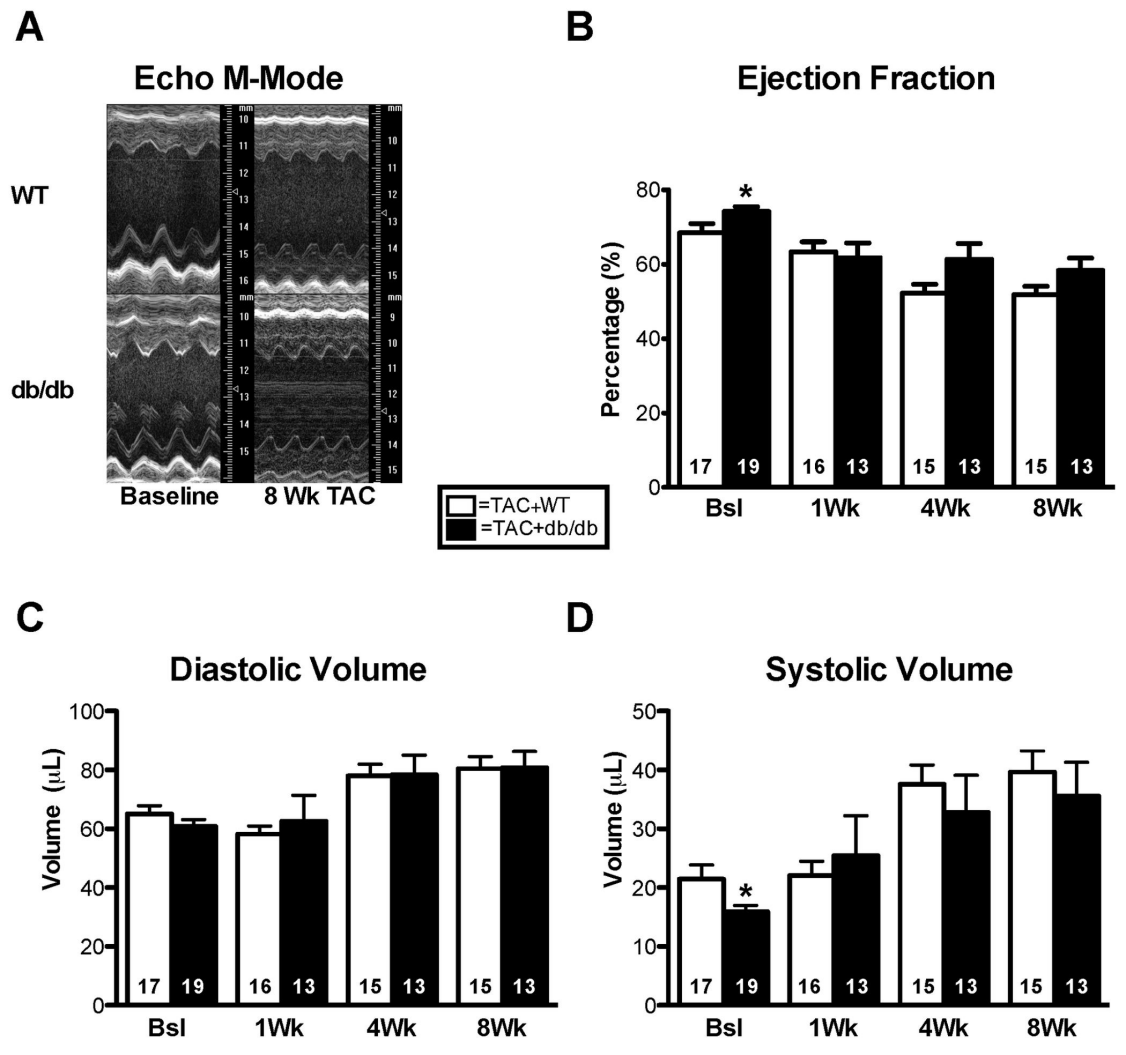
Hyperglycemia does not affect progression of pressure overload induced cardiac dysfunction. (**A**) Long axis m-mode utilizing the Vevo 770 imaging system. (**B**-**D**) Ejection fraction was not significantly changed from control groups through eight weeks of TAC. Diastolic and systolic volumes did not differ between the TAC+db/db and the TAC+WT groups. Results are expressed as means ± S.E., *, p< 0.05.

 After finding no differences between the ND and HFD models, we elected to go with a more robust model of hyperglycemia. To this end we used db/db mice, which display robust hyperglycemia and obesity. We subjected two cohorts of db/db mice and their heterozygous littermates to permanent coronary ligation, myocardial I/R, or TAC. db/db mice subjected to permanent coronary ligation had significantly increased mortality, also seen by Greer et al conducting similar experiments [33], resulting in decreased group sizes to such an extent that we discontinued those studies. Instead, we subjected db/db mice (n=10) and their heterozygous littermates (n=10) to 40 minutes ischemia and 5 days reperfusion. There were 5 mortalities in the db/db group compared to 2 in the WT group, though this difference in mortality was not significant. db/db mice weighed significantly more than WT (40.6±1.0 g vs. 23.1±1.5 g, p0.05). Echocardiography showed increased systolic and diastolic diameter and decreased ejection fraction and fractional shortening compared to their heterozygous controls (Figure 5A-D). Such differences were likely secondary to the increased infarct size we have already reported [18]. db/db mice and their heterozygous littermates were subjected to transverse aortic constriction and were assessed via echocardiography at pre-op, 1, 2, 4, and 8 weeks. Body weights and blood glucose were both significantly elevated in the db/db group. No difference was seen in survival rates (Table 3). Although a baseline difference in ejection fraction and end systolic volume was observed between the groups, there were no echocardiographic differences between the groups following TAC (Figure 6A-D).

**Table 3:** Left ventricular diameters, wall thicknesses, and fractional shortening of db/db TAC group; additionally, body weight, blood glucose, and survival data.

**Time Point**	**Group**	**LVIDd (mm)**	**LVIDs (mm)**	**LVPWd (mm)**	**LVPWs (mm)**	**LVAWd (mm)**	**LVAWs (mm)**	**FS (%)**	**Body Wt (g)**	**Glucose (mg/dL)**	**Survival (Fraction)**
**Pre-Op**	**WT TAC**	3.9±0.1	2.4±0.1	0.7±0.0	1.1±0.0	0.7±0.0	1.1±0.0	38±2	26.7±0.3	n/a	n/a
	**db/db TAC**	3.8±0.1	2.2±0.1	0.7±0.0	1.2±0.0	0.7±0.0	1.2±0.0	43±1	44.9±0.6*	n/a	n/a
**1 Week**	**WT TAC**	3.7±0.1	2.4±0.1	0.9±0.0	1.3±0.1	0.9±0.0	1.3±0.0	34±2	n/a	n/a	n/a
	**db/db TAC**	3.8±0.2	2.5±0.2	0.8±0.1	1.2±0.1	0.9±0.0	1.3±0.1	33±3	n/a	n/a	n/a
**4 Week**	**WT TAC**	4.2±0.1	3.1±0.1	0.9±0.0	1.2±0.0	0.9±0.0	1.3±0.0	27±1	n/a	n/a	n/a
	**db/db TAC**	4.2±0.1	2.8±0.2	1.0±0.0	1.4±0.1	1.0±0.0	1.4±0.0	34±3	n/a	n/a	n/a
**8 Week**	**WT TAC**	4.2±0.1	3.1±0.1	0.9±0.0	1.3±0.0	1.0±0.0	1.3±0.0	27±1	29.5±0.5	202.±18	15/17
	**db/db TAC**	4.2±0.1	2.9±0.2	1.0±0.0	1.4±0.0	1.1±0.0	1.5±0.0	31±2	54.3±0.9*	326±44*	13/19

* p<0.05 vs. WT TAC

### Hypoinsulinemia does not Alter TAC Induced Heart Failure

**Figure 7:**
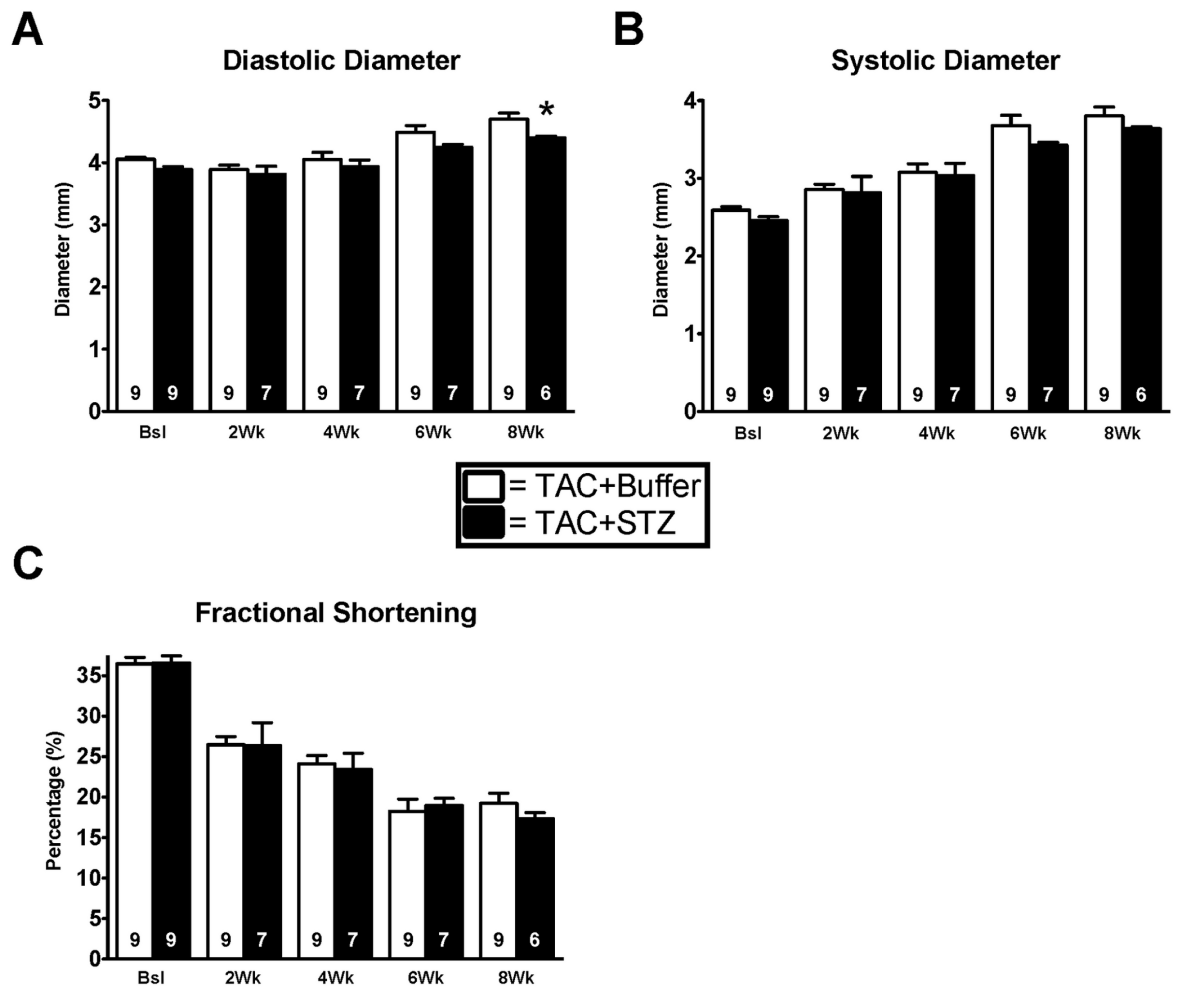
Pressure overload is not exacerbated by hypoinsulinemia via streptozotocin treatment. (**A**) Fractional shortening remained unchanged between the groups through eight weeks of TAC. (**B**-**C**) Diastolic and systolic diameters were assessed via long axis m-mode echocardiography on the Acuson Sequoia C512 imaging system. No change between TAC+Buffer and TAC+STZ treated mice was observed through eight weeks of TAC. Results are expressed as means ± S.E., *, p< 0.05.

We also utilized the streptozotocin induced hyperglycemic-hypoinsulinemia model to test whether cardiac dysfunction during TAC could be attributed to hypoinsulinemia. To test this hypothesis, we subjected 10-week-old C57BL/J6 mice to serial streptozotocin injections. After treatment, mice were subjected to TAC and cardiac function was assessed via echocardiography at pre-op, 2, 4, 6, and 8 weeks. After 8 weeks, survival rates were 6/9 in the streptozotocin treated group and 9/9 in the control group (p=NS) and body weights were not significantly altered (24.8±0.5 g vs. 26.4±0.9 g, p=NS). Streptozotocin injected mice had 3 fold higher blood glucose levels (583±18 mg/dL vs. 185±12 mg/dL, p<0.05). There were no differences in diastolic or systolic diameters through 6 weeks of TAC between the groups; however, at the 8 week time-point there was a significant difference in end diastolic diameter between streptozotocin treated mice and vehicle treated mice (Figure 7A-B). Despite this difference, there were no changes in ejection fraction and fractional shortening between the groups throughout the 8 weeks of TAC (Figure 7C).

## Discussion

Here, we show that increased fat intake, either before or after myocardial infarction, does not exacerbate cardiac dysfunction. We also show that severe hyperglycemia or hypoinsulinemia confer no deleterious cardiac effects during pressure overload induced heart failure. We utilized two different approaches to test the hypothesis that a HFD exacerbates progression of infarct-induced heart failure. The first method was to infarct mice that had been fed a ND and determine if a HFD post-infarct had any aggravating effect on progression of infarct induced heart failure; there was no change in cardiac function between the post-fed groups throughout the four-week protocol. The second approach was to pre-expose the mice to a high fat diet for 6 weeks before infarction in order to determine if a HFD predisposed mice to exacerbated cardiac dysfunction during infarct induced heart failure. The pre-fed mice, instead, showed no altered cardiac function compared to ND mice. Echocardiographic and pressure volume loop data were acquired and showed no significant differences in any of the measured endpoints.

 These findings challenge universal acceptance of this model and suggest the possibility that a HFD confers no greater risk of cardiac dysfunction once heart failure is induced via infarct. While these collective results may be somewhat surprising to many – because obesity and diabetes have been shown to be leading risk factors for the development of heart disease – they are, however, consistent with a limited number of recent reports. For example, Khairallah et al [11] showed improved mitochondrial function from supplementing mice with docosahexaenoic acid. Another group reported that a high fat diet post-infarction in rats does not exacerbate left ventricular dysfunction [9].

While we observed that feeding mice a diet high in fat caused elevated blood glucose but no cardiac dysfunction, we decided the next logical step was to determine if gross hyperglycemia or hypoinsulinemia had any adverse effect on cardiac function in our *in vivo* models. To this end, we used the db/db and STZ models to test this hypothesis and found that severe hyperglycemia in db/db or STZ-treated mice showed no greater cardiac dysfunction during pressure overload than their heterozygous littermates. It is, however, important to note that we did observe differences in cardiac function between db/db and wild-type mice following ischemia/reperfusion. We posit that the exacerbated cardiac dysfunction in the db/db mice was a result of their previously documented larger infarct sizes (10). In addition to their greater susceptibility to ischemia/reperfusion injury, the extreme level of genetically induced obesity and hyperglycemia may also contribute to the exacerbated dysfunction. Unfortunately, survival in the db/db mice following permanent coronary ligation precluded any head-to-head comparisons with the HFD results. Nevertheless, the result of our experience with the db/db mice supports the contention that the db/db model of diabetes exacerbates ischemia/reperfusion induced cardiac dysfunction.

 Interestingly, the current results indicate that a HFD desensitizes cardiac mitochondria to calcium induced swelling, which might imply that a HFD could exert a pro-survival effect via mitochondria in mice; this, of course, may not be the case in humans. Yet, we hypothesize that the HFD is potentially altering mitochondrial membrane phospholipid composition [7], which may be delaying the opening of the mitochondrial permeability transition pore. It is possible that such a beneficial effect on mitochondrial preservation could partially offset other deleterious effects of high-fat feeding, though it is unclear why such a potential effect may not have been present in earlier studies. It is also important to note that there were potential differences in survival, though they were not statistically significant.

 As with any study, particularly a largely negative study, there are important limitations to address. It is possible that particularly extended periods of HFD may exacerbate cardiac dysfunction during infarct-induced heart failure. The data from the extended HFD feedings rule out the possibility that the effects were simply delayed. Indeed, when naïve mice were fed two varieties of high fat diet (HFDM and HFDL) for a period of 18 or 28 weeks there was no significant reduction in cardiac function. In addition, any potential transient cardio-depressant effects of general anesthesia were avoided by performing conscious echocardiography in the 28-week group of mice. For the shorter durations of HFD, our selected time of six weeks was comparable with much of the current literature because this provides sufficient time for induction of increased triglycerides, ceramides, and free fatty acids, which are hallmarks of a diet induced obesity [7,34-36]. To address the issue of similar ingestion of food, mice were weighed prior to surgery and prior to sacrifice. HFD mice were significantly heavier than the ND controls at both time points. Most importantly, neither group lost a significant amount of weight over the course of the study, suggesting (but not conclusively demonstrating) that no difference in food intake occurred. We also assessed infarct size and found no difference between the groups after 24 h of coronary ligation. Infarct size compared to left ventricular area was 39±3% for the ND group and 42±2% for the HFD group (p=NS). Thus, differences in infarct size do not explain our results in the infarcted HFD groups.

 Again, we did not observe significant differences in survival in the HFD groups; however, some could argue that there was a potential trend toward worsened survival in the HFD groups. In addition, it is possible that diastolic dysfunction occurred prior to systolic dysfunction. Clearly, the infarct models represent primarily systolic defects; yet, the long-term HFD could present such an opportunity to evaluate defects in diastolic dysfunction. Assessment of diastolic function is difficult in humans, much less mice. Nevertheless, our estimations of E/A ratios showed no obvious diastolic defects in the 18wk HFDM or HFDL groups (data not shown).

In addition, some could also argue that permanent coronary ligation might be too severe a model, therefore masking any difference that could potentially be evidenced in more mild cardiac insult. To address this issue we used the TAC (transverse aortic constriction) model and found no differences in cardiac function after TAC in the genetically induced obesity (db/db) or hyperglycemic (STZ) groups. Lastly, there may be other changes in vascular function and/or molecular signaling that contribute to a pathophysiological aspect of HFD/diabetes, which do not impact cardiac function. Despite several potential limitations, we are confident that HFD alone may not be sufficient to produce systolic dysfunction in mice.

In summary, we show that mice exposed to a HFD before or after infarct-induced heart failure exhibit no exacerbation of cardiac dysfunction. We also show that mice with hyperglycemia or hypoinsulinemia exhibit no exacerbated cardiac dysfunction when subjected to transverse aortic constriction. Even long-term HFD (regardless of source) was unable to recapitulate defects in cardiac function reported in the literature. Our findings, in part, serve as a cautionary note when using certain mouse models to recapitulate complex human pathophysiology. It is likely that feeding rodents a HFD alone unreliably phenocopies humans consuming a western diet. Instead, a high fat combined with high sucrose diet may more accurately mimic a contemporary western diet [34,37,38]. At a minimum, there are potentially other unknown environmental factors that could contribute to the HFD-induced cardiac pathology reported by others.
