# Correction: High Fat Feeding in Mice Is Insufficient to Induce Cardiac Dysfunction and Does Not Exacerbate Heart Failure

**DOI:** 10.1371/journal.pone.0113944

**Published:** 2014-11-12

**Authors:** The PLOS ONE Staff

There is an error in the second sentence of the Methods section. The correct sentence is: Both diets were purchased from Research Diets Inc. HFD (Cat #:D12492) consists of 20% protein, 20% carbohydrate, and 60% fat.

There is an error in the fifth sentence of the Methods section. The correct sentence is: For long-term HFD feeding, 6-week-old male C57BL/6 mice were placed on a HFD (60% cal) derived from either lard (Harlan, TD.06414; HFDL) or milk (Harlan, TD.09766; HFDM), or low fat diet (Harlan, TD.06416; LFD) for 30-33 weeks.
